# Predicting work engagement among young adult cancer survivors: A moderated mediation model

**DOI:** 10.3389/fsoc.2023.1030518

**Published:** 2023-03-06

**Authors:** Siti Nur Syuhada Musa, Siti Raba'ah Hamzah, Zulaiha Muda, Soaib Asimiran, Steven E. Krauss

**Affiliations:** ^1^Faculty of Educational Studies, Universiti Putra Malaysia, Serdang, Malaysia; ^2^Institute of Pediatric, Hospital Kuala Lumpur, Kuala Lumpur, Malaysia; ^3^Institute for Social Science Studies, Universiti Putra Malaysia, Serdang, Malaysia

**Keywords:** employee resilience, workplace social support, workplace spirituality, work engagement, young adult cancer survivors

## Abstract

**Introduction:**

Early research on cancer survivors was focused on exploring cancer treatments. More recently, attention has shifted to cancer survivorship research, focusing on cancer survivors as individual persons, including the multiple facets of survivors' quality of life but is inapplicable in the context of work-related role played in the young adult cancer survivors' lives. However, in recent studies on the outcomes of long-term survivorship, some of the main areas of cancer survivorship research revolves around employment issues of young adult cancer survivors. In the present study, the focus is given on the role of workplace spirituality as a mediator on the association of employee resilience and workplace social support on work engagement in a Malaysian setting, taking into consideration gender and age differences.

**Methods:**

Using a quantitative research paradigm, data were collected from 270 respondents at Pediatric Institute Kuala Lumpur Hospital. Data were analyzed using descriptive statistics and the Partial Least Square-Structural Equation Model (PLS-SEM) to test the direct, indirect, and mediation effects.

**Results and discussion:**

The findings revealed that workplace spirituality did mediate the influence of employee resilience and workplace social support on work engagement. The findings also indicated that gender and age moderated the association of employee resilience and work engagement *via* workplace spirituality.

## 1. Introduction

Cancer results in significant to economic burdens on health sectors, patients and society. Cancer has a serious psychosocial effect on patients' work-related concerns (Dusetzina et al., 2014; de Souza et al., 2016). Besides contributing to the workforce, being employed is important for the individual's wellbeing financially and socially. Gainful employment gives the individual a sense of accomplishment. Unfortunately, withdrawal from work participation is often associated with cancer survivors (Chow et al., 2015). Globally, improvements in early detection and effective treatments of cancer have led to a high survival rate. Indeed, the rate of cancer survival has recently been increasing in Malaysia as well. Malaysian study on cancer survival (MySCan) in 2016 reported that the death from the government hospital (12.6%) and private hospital (26.7%). The study also shows an increasing trend from 11.3% (2007) to 12.6% (2016) with around 37,000 per year with firstly diagnosed with cancer and it was estimated to increase to 55,000 cases in the year 2030. Referring to Malaysia's National Cancer Registry Department report in 2018 around 21% of the overall sample, there were higher in women and younger patients (15–44 years old) for the survival rate (National Cancer Registry Department, and National Cancer Institute, 2018).

Existing literature focused on work limitations for cancer survivors (Mehnert et al., 2013; van Maarschalkerweerd et al., 2019). However, such a research focus might spark misconceptions of employers toward the ability of cancer survivors to function adequately at the workplace and might also lead to negative perceptions of the impacts of cancer treatments and cancer in relation to work (Grunfeld et al., 2010; Fitch and Nicoll, 2019). Cancer survivors tend to encounter chronic fatigue and emotional problems at the workplace (Becker et al., 2015). It is also reported in previous studies that decreased employment quality (Marques et al., 2019), in the shape of lower levels of job satisfaction and job performance decreased organizational commitment (Gonzalez et al., 2018). As a result, cancer survivors often face negative perceptions of their ability and contribution to their organizations. In the context of the workplace, the young adult cancer survivors' daily lives, which include their wellbeing, engagement, and perceived competency in performing their jobs are yet to be explored (Mehnert, 2011). Moreover, there also seemed to be an insufficient study on predicting these concepts related to employed cancer survivors' gender and work engagement. Apart from that, there is a link between employee engagement and demographics such as age (Gupta et al., 2016; Hamzah et al., 2020).

In this study, work engagement among young adult cancer survivors in Malaysia was investigated. Cancer survivors in their adolescence and young adulthood have distinct, understudied psychosocial support needs that are frequently disregarded by pediatric and adult oncology care models (Hall et al., 2011). Nevertheless, they face challenges including post treatment-related effects like older cancer survivors (Oshio et al., 2018), substantial impact on behavior, health and stress anxiety toward recurrence, increasing psychosocial needs burden, and difficulty in assessing psychosocial services (McManus et al., 2008). Unique survivorship practices such as constraints in social support networks and feelings of uncertainty may impact survivors with regard to how they manage their lives after surviving cancer.

Individual and work-related factors foster work engagement behavior. Engaged employees often go the extra the mile. Thus, it is important for practitioners to identify factors that promote work engagement behavior. Previous researchers have also embarked on the exploration of the role of personal factors (especially resilience) that can enhance work engagement behaviors (Malik and Garg, 2020; Pramanik et al., 2020). Resilient employees exhibit positive emotions and are quick in adapting to adversities and uncertainties especially in the business environment. In the present study, employee resilience is defined as employee capability, encouraged and assisted by the organization, to use resources and to persistently adapt and succeed at work, even when faced with difficult circumstances (Näswall et al., 2019).

In response to calls in the literature for an examination of potential antecedents and mediators of work engagement (Albrecht, 2013), this study was conducted to investigate whether individual and work- related factors might have a positive relationship with work engagement. One plausible mechanism through which individual and work-related may exert these positive effects on work engagement is the involvement of psychological processes. The first to coin the work engagement concept, Khan (1990) postulates that an employee will be engaged when he or she is psychologically attuned to conditions at the workplace, i.e., when psychological meaningfulness acts as a mediator on work engagement. Work that is meaningful and purposeful has also been identified as the most important dimension of workplace spirituality (Ashmos and Duchon, 2000). Albrecht (2013) suggests that psychological mechanisms (workplace spirituality) can explain how and why the provision or experience of either individual or personal factor (employee resilience) and work-related factors (workplace social support and QWL) results in increased engagement.

An important mechanism to bolster engagement at the workplace is through workplace spirituality (Singh and Chopra, 2018) which focuses on the humanistic aspect of work. Indeed, workplace spirituality has emerged as a positive organizational scholarship field (Lavine et al., 2014). Workplace spirituality creates wholeness and contentment (Dent et al., 2005) such that employees can find purpose and meaning in their work; they can express their complete selves at work and feel connected with others at the workplace (Kinjerski and Skrypnek, 2004). Workplace spirituality is postulated to enhance work engagement (Benefiel et al., 2014). In addition, it is recommended that organizations create an environment that is conducive for employees to experience spirituality so that the level of work engagement can be raised (Breytenbach, 2016). When employees' hearts and minds are captured at the workplace, the organization can be assured of totally engaged employees (Murray and Evers, 2011).

The present study is motivated by recent findings on workplace spirituality and work engagement by Milliman et al. (2018), who recommended more research to explore and determine the underlying human factors such as motivation to encourage employees to be more engaged at the workplace. However, only a few studies and the existing literature fails to offer convincing empirical evidence about the mechanisms through how employee resilience and social support affect work engagement among young adult cancer survivors. In this study, the predictors of work engagement among a sample in Malaysia were investigated. Secondly, the mediating role of workplace spirituality was examined. Lastly, the differences in the mediation-moderation model according to gender and age were scrutinized.

## 2. Theoretical framework

The present research grounds on Self Determination Theory (SDT) (Deci and Ryan, 1985) and its extended paradigm of work engagement to propose a conceptual framework. According to Deci and Ryan (1985), you must engage in an activity with a complete sense of desire, selecting, and personal endorsement before you can be said to be self-determinant. The three types of psychological needs are relatedness, competence, and autonomy (Ryan and Deci, 2000, 2017). The three psychological requirements do, in fact, play a significant role in growth, adjustment, and wellness across cultural boundaries, with significant consequences for fundamental areas of motivation, practical applications, and even general societal policies (Gillison et al., 2019). The basic psychological needs indeed may improve the level of resilience. According to research by Skinner et al. (2013), all three of a person's basic needs are positively connected with adaptive behaviors including planning, asking for help, being self-engaged, being committed, etc. For cancer survivors, competency specifically predicted future emotional resilience and coping mechanisms in regard to job engagement; relatedness was favorable to a solid workplace relationship; the term “autonomy” refers to the capacity to control one's own behavior and activities in the pursuit of stated objectives (Ryan and Deci, 2000). This study builds on these concepts by stipulating how employee resilience, workplace social support and Workplace Spirituality can lead to greater work engagement.

### 2.1. Employee resilience and work engagement

Past study has shown that individuals who have experienced much adversity in their lives tend to acquire better coping skills and thus become more resilient. According to Oshio et al. (2018), resilience as a personality characteristic can moderate the negative effects like stress and promotes adaptation. In this regard, employee resilience is related to the capacity of the employees, assisted, and supported by the organization management to utilize resources for continual adaptation, helping the employee to positively cope in response to change over the work environment (McManus et al., 2008). Employees who are resilient persevere in the face of difficulties, display confidence in their skills, and motivate others to work more enthusiastically (Hodliffe, 2014; Cooke et al., 2016). Resilience has been characterized as an employee's behavior that fosters work engagement while they are working for a company by utilizing their own resources related to their jobs.

Past research revealed that highly resilient individuals are capable of coping and adapting to adversity such as stress at the workplace (Hodges et al., 2008). Employees that are resilient are better equipped to cope with the negative impacts of work stress (Piotrowski et al., 2021). Malik and Garg (2020) argued that resilience acts as a trigger point for cultivating positive feelings among employees. Specifically, when employees express their positive reactions, it prompts increased psychological resources which encourage resilience to cultivate work engagement. In addition, Nieto et al. (2022) emphasized the role of resilience in predicting work engagement. They found that when resilient employees faced adversity, they could successfully cope, adapt, and recover due to a high level of work engagement. Building on these findings it is hypothesized that:

Hypothesis 1 (H1). Employee resilience significantly influence work engagement.

### 2.2. Workplace social support and work engagement

Kiema-Junes et al. (2020) found that employees' work engagement is significantly influenced by social support at work. The research involved two contexts of social support, specifically supervisory and collegial support, both of which showed similar associations. In another related study involving personnel training services sector, social support positively predicted work engagement (Bonaiuto et al., 2022). The study found that an increase in supervisor social support can boost work engagement. Supervisory support is an important factor that can help ensure the attainment of work goals and reduce the stress associated with high job expectations, resulting in increased work engagement (Schaufeli and Bakker, 2004). Likewise, Wolter's research found that support from supervisors and coworkers, job resources and personal resources were all frequently related to predicting work engagement (Wolter et al., 2019). Moreover, this study also confirmed social support from supervisors and coworkers promotes work engagement.

Another factor is social support can also be effective in promoting engagement. From the perspective of the social exchange theory, when employees feel supported by their supervisors, they are more likely to feel indebted and obliged to give back to the organization resulting in increased work engagement (Cropanzano and Mitchell, 2005). Furthermore, employees who feel that they have been rewarded with a conducive environment because of their roles in the organization would usually respond by becoming more energetic and more absorbed in their work (Saks, 2006). Based on the literature, the hypothesis is formulated below:

### 2.3. Workplace spirituality as a mediator

A spiritually nourishing workplace recognizes the importance of employees' inner lives, which are nourished by meaningful work, a sense of community, and alignment with the company's values (Ashmos and Duchon, 2000). Previous studies have stated a correlation between workplace spirituality and work engagement (Roof, 2015; Milliman et al., 2018). According to a past study by Petchsawang and McLean (2017), they stated workplace spirituality is necessarily associated with work engagement among employees in Thailand. The findings indicate that when people describe their work to be meaningful, they were more inspired, passionate, and committed to their work. More recently, researchers have started to study the mediating effects of workplace spirituality on work engagement. The capability of workplace spirituality as a mediator of work engagement might lie in its relationship to meaningful work. Jena and Pradhan (2018) found that workplace spirituality uniquely impacted the loyalty and commitment of the employees, and they feel more effectively attached to their organization. Furthermore, it will reinforce their devotion to work, giving them a strong sense of contentment (Izak, 2012). Other studies have identified the mediating effects of spirituality in the workplace on deviant behavior (Haldorai et al., 2019) employee's emotions and ethical behavior (Naseer et al., 2019); task significance and performance (Allan et al., 2016); and self-esteem and wellbeing (Orkibi and Bar-nir, 2015).

As for the present study, those who overcame challenging cancer disease and treatment processes such as major surgery and radiation have strong spiritual attributes (Garduño-Ortega et al., 2021) and spirituality in particular facilitated patients cope with the disease (Acquaye et al., 2022). This is because spirituality provides a means of dealing with problems and reducing negative feelings and the negative consequences of stressful activities (Borges et al., 2017). When individuals trust in powerful energy, they lean on it; therefore, they have less fear and can manage any unfortunate events. The level of spirituality remains elevated even after a subsequent diagnosis and treatment (Hoseini et al., 2016; Toledo et al., 2020). Through intuitive understandings and insights, spirituality helps people find the meaning of life amid adversity and over-come adversity (Yoon, 2009). When psychological capital such as optimism, resilience, self-efficacy, and hope is achieved by workers, the need for satisfaction from work is met. This satisfaction drives an enhanced sense of engagement in the workplace (Albrecht, 2013). Hence, people who have overcome adversity and are resilient, such as cancer survivors, by seeking purpose in life and attempting to reinterpret them, have an elevated workplace spirituality rate than the average employees (Jin and Lee, 2019). Despite the growing interest in workplace spirituality as a mediator of engagement, gaps remain. Thus, this study proposes workplace spirituality as an underlying mechanism between resilience and work engagement.

Next, workplace spirituality will mediate the relationship between workplace social support and work engagement. Nawrin (2018) found that meaningful work (dimension of workplace spirituality) partially mediated job resources and work engagement among bankers. Job resources such as social support autonomy, performance feedback, opportunities for learning and development, and task significance were found to be strong predictors of work engagement. Indeed, acts of kindness toward others stimulate reciprocal relationships with co-workers that result in increased engagement at work (Adnan et al., 2020; Saks, 2021).

Countless studies have been conducted and explored workplace spirituality as a mediator on the association between resilience and workplace social support toward work engagement. Thus, the objective of the present study is to bridge the gap in the literature by investigating the mediation effect of workplace spirituality between the predicting variables. The following hypotheses are therefore stated:

Hypothesis 3 (H3). The positive relationship between employee resilience and work engagement is mediated by workplace spirituality.
Hypothesis 4 (H4). The positive relationship between workplace social support and work engagement is mediated by workplace spirituality.

### 2.4. Age and gender as moderators

Apart from investigating the role of workplace factors on the work engagement of young adult cancer survivors, the moderation effects of age and gender are also studied. The social roles between men and women are different; thus, they behave inversely in the workplace (Trzebiatowski and Triana, 2018). They are different in terms of productivity and earnings according to the job description and job sector. In several studies, gender differences have been found to impact work engagement (Ling Suan and Mohd Nasurdin, 2016; Liu et al., 2017; Ghosh et al., 2019; Khodakarami and Dirani, 2020). However, previous studies also reported non-significant difference between gender and work engagement (Topchyan and Woehler, 2020). For example, Hartman and Barber (2020) found no statistically significant difference between occupational self-efficacy and work engagement between men and women. Based on the present report on gender in-consistencies in work engagement, therefore a shred of evidence that gender moderate employee resilience and social support in relation to workplace spirituality and work engagement. A better understanding on these relationships would be valuable to organizations in their attempts to develop appropriate strategies for retaining employees.

Previous researchers have suggested that age-related differences in work attitudes and behaviors may be a result of psychosocial changes (e.g., social role changes) as well as biological aging (Jimenez, 2020). Taken together, age is also an important demographic variable in explaining the variation of work engagement (Boyraz et al., 2012; Douglas and Roberts, 2020). Jones and Harter (2005) suggested that age possibility turns into a moderator on the association between employee engagement and turnover intention. The moderating role of age has also been shown to help in amplifying the explanatory power of work engagement (Meyers et al., 2019). Further, younger employees have shown an experience of lower levels of work engagement in comparison to middle- aged and older employees.

### 2.5. Research framework

**Figure 1:**
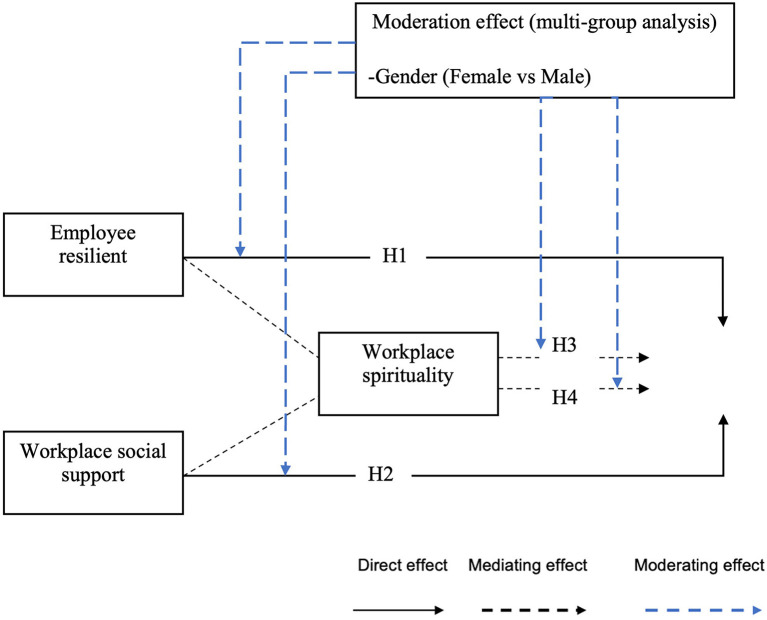
Research framework.

The present study investigates the correlation between employee resilience and workplace social support on work engagement. Further, by investigates the role of mediating the effect of workplace spirituality among respondents. The moderation effects in the context of age and gender are further explored (Figure 1).

## 3. Materials and methods

### 3.1. Sampling

The study sample consisted of 270 young adult cancer survivors in Malaysia. The selected respondents had been diagnosed with all types of cancer and were between 18 and 30 years of age at the time of collecting the data. The study respondents are inclusive of young adult cancer survivors who are provisionally registered for follow-up sessions at the Pediatric Clinic in Kuala Lumpur Hospital. G- power analysis was applied to estimate a sample size of 80% and an effect size of 0.15 (Cohen, 2013). The Pediatric Institute is a major referral center, while HKL is the largest public hospital in Malaysia. The current study was approved and granted by Medical Ethics Committee and National Medical Research Registry (NMRR18-85-40225-IIR) Ministry of Health Malaysia.

### 3.2. Data collection procedure

Surveys were completed at the preferred place and time of each respondent. Most of the sessions were conducted at the hospital following participants' medical appointments. Data collection was carried out in 1 year period. Prior to completing the survey, all subjects gave their written informed consent that their participation was voluntary and were briefed that all data would be treated confidentially. Following completion of the survey, a small souvenir was given to the respondents as a token of appreciation for their participation. The survey was presented in both Malay and English. As the original measures were in English, translation was carried out using back-to-back translation procedures to achieve semantic equivalence.

### 3.3. Measures

In measuring work engagement, this study utilized the UWES-17 scale that was developed by Schaufeli et al. (2002); which consisted of three dimensions with 17 items and used a seven-point scale that ranged from 0 to 6 (never to every day) and the composite reliability (CR) was 0.934, as shown in Table 1. Employee resilience was measured using the 12-item Employee Resilience Scale (EmpRes) which was proposed by Näswall et al. (2019). The items used a seven-point scale ranging from 1 to 7 (never to almost) and the CR was 0.924. Workplace social support consisted of two dimensions, namely, supervisor support and co-worker support. Workplace social support refers to cancer survivors' perception that their supervisor and co-workers value their contributions, compliment them, and genuinely care about their wellbeing (Haynes et al., 1999; Eisenberger et al., 2002). In the current study, Eisenberger's supervisor support scale consisting of four items, and Haynes' co-worker support scale consisting of three items were used. It consists of seven items on a five-point Likert scale of 1 (strongly disagree), 2 (disagree), 3 (not agree), 4 (agree) and 5 (strongly agree). The CR for workplace social support was 0.875. Spirituality at work was measured using SAW by Ashmos and Duchon (2000) and Milliman et al. (2003) consisting of 21 items on a seven scale from 1 (never) to 7 (always) with three subscales, which are sense of community, the alignment of core values and meaningful work. CR values for workplace spirituality were 0.948.

**Table 1:** Measurement model assessments (validity and reliability).

		**First iteration**				**Final iteration**			
		**Loading**	**AVE**	**CR**	**Alpha**	**Loading**	**AVE**	**CR**	**Alpha**
Work engagement	1	0.735	0.464	0.935	0.925	0.744	0.507	0.934	0.923
	2	0.691				Delete			
	3	0.788				0.788			
	4	0.788				0.799			
	5	0.814				0.822			
	6	0.783				0.799			
	7	0.738				0.737			
	8	0.834				0.833			
	9	0.822				0.823			
	10	0.815				0.814			
	11	0.815				0.815			
	12	0.732				0.782			
	13	0.698				Delete			
	14	0.484				Delete			
	15	0.764				0.846			
	16	0.780				0.836			
	17	0.576				Delete			
Employee resilience	1	0.688	0.577	0.924	0.908	0.688	0.577	0.924	0.908
	2	0.721				0.721			
	3	0.793				0.793			
	4	0.804				0.804			
	5	0.774				0.774			
	6	0.807				0.807			
	7	0.807				0.807			
	8	0.770				0.770			
	9	0.660				0.660			
Workplace support	1	0.657	0.483	0.862	0.810	0.694	0.544	0.875	0.825
	2	0.733				0.727			
	3	0.560				Delete			
	4	0.771				0.802			
	5	0.875				0.874			
	6	0.884				0.885			
	7	0.835				0.835			
Workplace spirituality	1	0.694	0.489	0.952	0.947	Delete	0.502	0.948	0.942
	2	0.749				0.756			
	3	0.802				0.792			
	4	0.765				0.764			
	5	0.763				0.788			
	6	0.730				0.779			
	7	0.801				0.800			
	8	0.743				0.740			
	9	0.769				0.769			
	10	0.781				0.781			
	11	0.746				0.748			
	12	0.719				0.721			
	13	0.709				0.709			
	14	0.773				0.795			
	15	0.745				0.766			
	16	0.758				0.780			
	17	0.763				0.774			
	18	0.801				0.803			
	19	0.721				0.738			
	20	0.696				Delete			
	21	0.705				Delete			

## 4. Results

### 4.1. Demographic profile

Of the 270 young adult cancer survivors aged 18 to 30 years (57.8% of a younger group aged 18–25; 42.2% of an older group aged 26–30) participated in this study. Participation of female young adult cancer survivors was slightly higher at 51.5% of the total sample. As for the educational background in this sample, 1.5% held a Master's degree, 27.8% of the participants had a Bachelor's degree, 30.7% with a Diploma, and 39.8% had a Malaysian Certificate of Education (Higher and Lower). The data also revealed that 45.9% had permanent jobs, about 20.4% were temporarily employed, self-employed (25.6%) and part-time employees (8.1%). A higher percentage of their income was less than RM 3,000 monthly (73.7%) compared with respondents who earned RM 3,001–RM 5,000 (21.1%) or more than RM 5,000 (5.2%). Besides, 89.6% of the respondents had been diagnosed with cancer once, and about 10.4% of respondents were diagnosed more than once. For the types of cancer almost half of the respondents had leukemia (52.2%), followed by Hodgkin's lymphoma (10.7%), Wilms' tumor (5.9%) and ovarian cancer (3.7%).

Since the study hypotheses were directional, the study relationships were examined using variance-based structural equation modeling (Hair et al., 2017). To validate the research model, partial least squares structural equation modeling (PLS-SEM) was used (Hair et al., 2020). This analysis is applied at two stages: measurement model and structural model. The relationships between constructs and their accompanying indicator variables are represented by the measurement model, also known as the outer model. Next, the structural model analysis was conducted to confirm or reject the hypotheses as well as to understand the strength of the relationships between dependent and independent variables.

### 4.2. Measurement model

To measure the reliability and validity of the outer model, tests for internal consistency, convergent validity, and discriminant validity were carried out (Hair et al., 2017). Internal consistency was assessed using composite reliability values and each variable needed to exceed more than 0.7 (Hair et al., 2016; Henseler et al., 2016). The present study shows (Table 2) that composite reliability scores were between 0.80 and 0.91 which were above the recommended threshold of 0.70, thus indicating that the items used to represent the variables had satisfactory internal consistency reliability (Hair et al., 2017). The average variance extracted (AVE) value of each dimension indicates good convergent validity which is >0.5.

**Table 2:** The measurement model discriminant validity.

	**Employee resilience**	**Social support**	**Work engagement**	**Workplace spirituality**
Employee resilience				
Social support	*0.855*			
	CI 0.90			
	(0.791, 0.850)			
Work engagement	*0.750*	*0.740*		
	CI 0.90	CI 0.90		
	(0.638, 0.853)	(0.631, 0.817)		
Workplace spirituality	*0.676*	*0.697*	*0.669*	
	CI 0.90	CI 0.90	CI 0.90	
	(0.559, 0.770)	(0.548, 0.784)	(0.551, 0.747)	

Italic values have discriminant validity with value < 0.9; ▄ indicate the same variable.

Discriminant validity assesses how different the constructs in the model are from one another, and thus captures a single unique phenomenon (Hair et al., 2017). The Heterotrait-Monotrait ratio (HTMT) was applied to analyse the measurement model's discriminant validity. As suggested by Gold, when the correlations between the examined con-structs are < 0.9, a measuring model has appropriate discriminant validity, as indicated by the italic values in Table 2 (Gold et al., 2001). The analysis also included the lower (below 5%) and the higher (above 95%) ratio of the confidence interval. For example, the confidence levels for the constructs ranged from 0.638 to 0.853 at a 90 per-cent bias-corrected bootstrap confidence interval. This indicates sufficient discriminant validity between the study measures.

### 4.3. Structural model

The validity of the model was examined by assessing collinearity, significance, and relevance of the structural model path coefficients, coefficient of determination (R^2^), and predictive relevance (Q^2^) through structural model analysis. To examine the collinearity statistics, we used the variance inflation factor (VIF) (Mansfield and Helms, 1982). Table 3 shows that all VIF values were < 5, showing that collinearity among the structural model's exogenous constructs was not at a critical level (Hair et al., 2017). Nitzl et al. (2016) provided guidelines for testing mediation in PLS-SEM which were then used to test the hypotheses. The use of traditional goodness-of-fit indices in variance-based SEM approaches has been found to be unsuitable. Hair et al. (2017) introduced standardized root mean square residual (SRMR) as a method to avoid model misspecification when testing model fit. For model fit, an SRMR value of < 0.10 is considered acceptable (Hu and Bentler, 1998). To examine the significance of the parameter estimates, we used bootstrapping analysis with 5,000 samples. With an SRMR value of 0.06, it was showed a good fit of data according to the structural model estimated.

**Table 3:** Hypothesis testing of the structural model.

**Hypothesis**	**Std beta**	***t*-value**	**2.5 CI LL**	**97.5% CI UL**	***f^2^***	**VIF**	***R* ^2^**	***Q* ^2^**	**SRMR**
H1: ER-WE	0.371	3.938^**^	0.193	0.544	0.123	2.569	0.568	0.296	0.006
H2: SS-WE	0.211	3.113^**^	0.067	0.336	0.042	2.433			
H3: ER-WPS-WE	0.109	3.768^**^	0.062	0.174	0.132				
H4: SS-WPS-WE	0.081	1.982^*^	0.026	0.195	0.074				

^*^p < 0.05, t-value >2.33 at ^**^p < 0.01.

ER, employee resilience; WE, work engagement; SS, social support; WPS, workplace spirituality; CI, confidence interval, LL, lower level; UL, upper level; f^2^, effect size; SRMR, standardized root mean square residual.

Results showed that all the hypotheses of this study are supported and are in line with the existing literature. This result supports H1 (β = 0.371, *t* = 3.938^**^) and revealed that employee resilience had an influence on work engagement. Next, workplace social support also had a significant influence on work engagement (β = 0.211, *t* = 3.113^**^), hence H2 was supported. The results for indirect effects showed that workplace spirituality mediated the relationship between employee resilience and workplace social support on work engagement. Table 3 presents the results of bootstrapping analysis.

Furthermore, the coefficient of determination score (*R*^2^) was assessed to predict and ex-plain the model's predictive accuracy. *R*^2^ is a combination effect of exogenous variables on endogenous variables (Hair et al., 2017). Table 3 shows an *R*^2^ value of 0.568, indicating that employee resilience, social support and workplace spirituality had a moderate effect on work engagement (Henseler et al., 2015) and explain 56.8% of the variance. The relationship between employee resilience and workplace spirituality showed the highest effect size (0.196; refer to Table 3). According to Hair et al. (2017), *Q*^2^ values are important for explaining the predictive relevance of a structural model. To evaluate the predictive relevance of the proposed model, *Q*^2^ values are derived by employing the blindfolding procedure with the cross-validated redundancy method (Stone-Geisser's *Q*^2^ value) (Geisser, 1974). A model has predictive relevance when a *Q*^2^ is greater than 0, otherwise it lacks predictive relevance (Henseler et al., 2015). In Table 3, the value of *Q*^2^ was 0.296. Thus, predictive relevance is present in this model.

### 4.4. Mediation effect

Next, the mediation effect of workplace spirituality (i.e., indirect effect) on the relationship between employee resilience and workplace social support in relation to work engagement was assessed. According to Zhao et al. (2010), full mediation occurs when the direct effect of a predictor is not significant with the insertion of a mediating variable. In the current study, the findings show that a partial mediation for employee resilience and work engagement, therefore supported H3. The results also show that the strength was reduced from ß = 0.371 (*p* < 0.001) to ß = 0.109 (*p* < 0.001), however remaining significant for the workplace spirituality (Table 4). Furthermore, for H4, there was a significant and positive effect on workplace social support and work engagement but partial mediation. The direct effect reduced from ß = 0.221 (*p* < 0.001) to ß = 0.081 (*p* < 0.05). The findings indicate that young adult cancer survivors who felt supported by supervisors and co-workers were more likely to experience greater interconnectedness with their respective workplaces, hence encouraging them to be more engaged at work.

**Table 4:** Moderation analysis for gender (female vs. male).

**Path**	**PC (female)**	**PC (male)**	**PC (F–M)**	**Permutation *p*-values**	**PLS-MGA *p*-values**
H1: ER-WE	**0.565** ^***^	0.179	0.386^**^	**0.035**	**0.023**
H2: SS-WE	0.187^***^	0.240^**^	−0.053	0.372	0.350
H3: ER-WPS-WE	**0.160** ^***^	0.054	−0.094^*^	**0.049**	**0.074**
H4: SS-WPS-WE	0.036^***^	0.134^***^	−0.098^*^	0.111	0.083

^*^p < 0.1.

^**^p < 0.05.

^***^p < 0.01.

PC, path coefficient; F, female; M, male; WE, work engagement; ER, employee resilience; SS, social support; WPS, workplace spirituality.

Bold values indicate the value have significant moderation.

### 4.5. Multigroup moderation analysis

PLS-SEM MGA was used to analyze the moderating roles of age and gender on the relationships of direct effect on work engagement and the indirect effect of workplace spirituality. The MGA algorithm of Smart PLS 3.3.2 was used to compare the groups. The results of the multigroup moderation analysis (MGMA) are presented in Tables 4, 5. A minimum confidence level of 95% was used for the analysis.

**Table 5:** Moderation analysis by age (younger vs. older).

**Path**	**PC younger**	**PC older**	**PC (Y–O)**	**Permutation *p*-values**	**PLS-MGA *p*-values**
H1: ER-WE	0.277^***^	0.449^***^	−0.172^**^	**0.027**	**0.049**
H2: SS-WE	0.286	0.144	0.142	0.172	0.153
H3: ER-WPS-WE	0.092^**^	0.161^***^	0.069^*^	**0.012**	**0.041**
H4: SS-WPS-WE	0.103	0.048**	0.055	0.233	0.195

^*^p < 0.1.

^**^p < 0.05.

^***^p < 0.01.

PC, path coefficient; Y, younger; O, older; ER, employee resilience; WE, work engagement; SS, social support; WPS, workplace spirituality.

Bold values indicate the value have significant moderation.

Next, we examine the moderation analysis for gender. We divided the group of respondents into male (*n* = 131) and female (*n* = 139). The results revealed a significant moderating effect for the relationship of gender. Therefore, H1 was supported, and the findings were confirmed only females (βdiff = 0.565, *p* < 0.01) was significantly influenced the effect of employee resilience on work engagement (βdiff = 0.386, *p* < 0.05). From the result, we infer that female young adult cancer survivors' resilience had a positive effect on work engagement. Next, the results for the mediating effect showed that workplace spirituality mediates the relations between employee resilience and work engagement and moderated by gender (βdiff = −0.094, *p* < 0.1). The mediating effect of workplace spirituality reduced the impact of employee resilience on work engagement (direct effect). This relationship was stronger for female respondents (β = 0.160, *p* < 0.01) than for males (β = 0.054, *p* < 0.01; refer to Table 4).

Table 5 shows that, there are significant differences for H1 and H3 with the *p*-values for the difference in path coefficients were all lower than 5%. Besides that, age has a moderating effect on the relationship between employee resilience and work engagement. The results further indicated significant differences for the younger and older groups (βdiff = −0.172), with the permutation test (*p* = 0.027) and the PLS-MGA (*p* = 0.049) below 5%. Bootstrapping results employed to assess the difference between the age groups' path coefficients showed that the older group (β = 0.449) had a stronger path coefficient than the younger group (β = 0.277; Table 5). Thus, the relationship was stronger for the older group. This implies that older cancer survivors tend to be more resilient and thus more engaged at work.

Likewise, for H3, the mediating effect of workplace spirituality on the relationship between employee resilience and work engagement was moderated by age (βdiff = 0.069, *p* < 0.1). The mediating effect of workplace spirituality reduced the strength of the relationship between employee resilience and work engagement (direct effect). This relationship was stronger for the older respondents (β = 0.161, *p* < 0.01) as compared to the younger respondents (β = 0.092, *p* < 0.05). However, the results also show that age did not moderate the relationships posited in H2 and H4.

## 5. Discussion

This study concludes that being a cancer survivor is not an excuse to be less engaged at the workplace. Employers should not expect poor work outcomes of cancer survivors. Cancer survivors can have positive characteristics and be just as engaged at work as that healthier employee. Therefore, the negative perception that cancer survivors are not engaged at the workplace should be discarded. Despite the growing focus on the importance of work engagement for young adult cancer survivors, this study is the first to evaluate a moderated mediated model after completing their cancer treatment. The current study revealed several important insights that could con-tribute to the body of knowledge on work engagement. Based on existing literature and past research, a comprehensive conceptual framework that combines key behavioral and demo-graphic factors was proposed and tested explaining work engagement, employee resilience, workplace social support, and workplace spirituality. This study also broadens past research findings by examining the determinants of work engagement among young adult cancer survivors. The mediating role of workplace spirituality on the relationship between employee resilience and workplace social support regarding work engagement is also examined. This study makes several contributions to the theoretical and managerial issues.

First, employee resilience and workplace social support have been validated as key predictors of work engagement. A direct correlation exists between employee resilience and work engagement. Resilience enhances cancer survivors' work engagement. The results corroborate Malik and Garg's (2020) study, which found that cultivating employee resilience aids a pathway for developing work engagement. Additionally, there is a positive relationship between employee resilience and work engagement (Black et al., 2017; Kašpárková et al., 2018). Thus, employees who have strong characteristics such as resilience tend to be more engaged at work. The effect of resilience on work engagement was significantly stronger for female cancer survivors as well as for relatively older cancer survivors. This is in line with the trend that women cancer survivors are more expected to experience work stress and burnout than it does men (Rees et al., 2015). Their psychological resilience enables them to respond positively to potentially stressful situations, thus protecting them from the potential negative effect of stressors (Fletcher and Sarkar, 2013; Rees et al., 2015). Resilience helps to alleviate the adverse effects of work stress for female cancer survivors, thus strengthening their work engagement as well.

Workplace social support is also found to significantly impact work engagement consistent with prior research findings (Geisler et al., 2019; Wolter et al., 2019; Kiema-Junes et al., 2020). Wolter et al. (2019) found that job resources like social support could promote work engagement internally and externally. Accordingly, the current study resonates with the work of Geisler et al. (2019), who reported on the importance of supervisor and co-worker support as contributors to employee work engagement. A better understanding on how young adult cancer survivors received more support from supervisors and co-workers could assist and improve their engagement with their work, regardless of their gender and age. Just as no significant differences were found between male and female respondents, differences in social support on work engagement were also found to be non-significant with regard to age.

As expected, employee resilience positively predicted workplace spirituality and Saks (2006) assert that certain personal characteristics have a close association with workplace spirituality. Previous studies have shown that the application of signature strengths at work was positively related to positive experiences at work as it could stimulate workplace spirituality (Harzer and Ruch, 2012). Findings of this study also suggest that gender and age of the cancer survivors, and the effect of employee resilience on workplace spirituality do not differ significantly. The present findings are also consistent with previous reports, mentioning that positive workplace social support contributes to workplace spirituality (De Carlo et al., 2020). This study concludes that when young adult cancer survivors had support from their supervisor and co-workers, their socio-emotional needs were met, and they were able to find greater meaning and purpose in their work. Furthermore, the atmosphere at the workplace was conducive to fostering spirituality and work engagement (Duchon and Plowman, 2005; Paul et al., 2020).

Finally, regarding indirect effects, findings of this study demonstrate that mediation effects exist on the relationships of employee resilience, workplace social support with work engagement *via* workplace spirituality. Workplace spirituality has partially mediated the relationship between employee resilience and work engagement. These results also confirmed with the past study which recommended workplace spirituality as a key mechanism that converts resilience to work engagement (Nawrin, 2018). A plausible explanation for this is that employed cancer survivors behaved in a resilient manner; they were able to build themselves up and were positive in the face of adversity, thus nourishing workplace spirituality. They were able to find meaning in their work and were more engaged. In other words, being engaged in work requires strong emotional state that can stimulate spirituality at the workplace such as seeking the purpose of work and connectedness with others, resulting in positive outcomes in work. The individual will be satisfied when he or she finds purpose and meaning in work, thus influencing individual resources and work engagement. In other words, for employed cancer survivors, a series of adversities related to their cancer and treatment improve their adaptability and help them cope with problems, and ultimately contribute to positive outcomes such as being more engaged at work. As a kind of power to enable individuals to interpret and respond more positively to challenges (Allport, 1968), workplace spirituality plays an important role in bolstering resilience that enhances work engagement. Thus, enhancing workplace spirituality is critical as it plays a mediating role in helping cancer survivors overcome challenges at the workplace, face psychological difficulties, become more resilient, and become highly engaged at the workplace. Furthermore, these findings also give an impact on researchers and practitioners to assess the different predictors.

Workplace spirituality indirectly affects work engagement when there is workplace social support. van Dick et al. (2008) identified job satisfaction as a form of meaningful work, a dimension of workplace spirituality dimension that mediates the relationship between job resources (supervisor and co-worker) and vigor, a key component of work engagement. It can be concluded that when cancer survivors come into the office with positive feelings, i.e., imbued by spirituality, they will be more engaged at the workplace. Besides that, the level of employees' work engagement improves when their work is perceived as being meaningful and purposeful. Simultaneously, when employees are given workplace social support, work engagement is also enhanced. In addition, theoretically, organizational support for employees' spirituality would result in employees having increased motivation, creativity, commitment, and work engagement (Osman-Gani et al., 2013). Besides, when the organization allocates more effort and attention to implementing interventions program in improving employee spirituality, they will be more engaged in their work (Saks, 2006).

From the analysis of mediation, the findings revealed a positive indirect effect on employee resilience and workplace social support on work engagement through workplace spirituality. Employee resilience and workplace social support foster workplace spirituality, whereby, both are positively associated with work engagement. Furthermore, the moderation analysis also showed that gender and age were influenced by employee resilience on work engagement. When these findings are put together, the “moderation is mediated,” meaning that, the indirect effect of employee resilience on work engagement through work-place spirituality depends on age and gender.

Based on the results, this study contributes to the literature in several ways. Most importantly, we framed our study in the context of testing Self Determination Theory (SDT) (Deci and Ryan, 1985) and its extended paradigm of work engagement to propose a conceptual framework. This study advances SDT by empirically demonstrating the implication of resilience in stimulating employees' engagement amid the work challenges resulting from cancer and treatment-related. The present findings also emphasize the SDT as a platform to explain transactions of positive relationships between individual factor (employee resilience) and environmental factors (workplace social support) where cancer survivors desire to develop their positive behavior leading to work engagement. The theory lends support to ideas that can guide the crafting of policies, practices, and situations to improve both work engagement and performance. Second, another noteworthy set of results is the significant role of workplace social support in linking work engagement through the lens of SDT. Though workplace social support has been widely explored in human resource development (HRD) studies, the present study highlights the important role it plays in the work engagement of Malaysian cancer survivors, thereby adding to the literature. Third, an apparently novel contribution of the study is the significant reciprocal indirect relations linking employee resilience and workplace social support toward work engagement *via* workplace spirituality. The identification of workplace spirituality as a mediating mechanism through which employee resilience and workplace social support are related to employee work engagement. Lastly, the findings of the study contribute to a better understanding of the concept of work engagement among employed cancer survivors in Malaysia. Previous studies of cancer survivors in Malaysia focused mainly on health-related outcomes. More recently, researchers have begun to explore the role of work among cancer survivors, focusing especially on work engagement as the key factor in attaining both individual and organizational goals.

## 6. Implication

Few previous studies have employed quantitative analysis to generate empirical data on the phenomena of work engagement among cancer survivors. In contrast, previous studies have relied mostly on qualitative approaches in exploring the experiences of cancer survivors in the workplace (Aguiar-Fernández et al., 2021; Torp et al., 2021). From a practical perspective, the study framework provides evidence to employers to shelve concerns about cancer survivors' capabilities, especially with regard to their work engagement. They should instead help these employees meet the aspirations of the organization. The findings of the present study revealed that the fundamental factors which directly influence cancer survivors' work engagement are resilience and work-place social support.

Through mediated relationships, employers should be able to organize the interventions program in promoting workplace spirituality among cancer survivors and the organization. Thus, we suggest that employers should not be hesitant to employ cancer survivors. Organizations especially HR managers should also rationally consider providing work that suits the ability of cancer survivors, i.e., work that does not involve too much physical effort. As human resource development focuses on competency development, it is in a position to attend to any issue that might possibly affect organizational performance and development, especially among employed cancer survivors. It is also suggested that HR professionals assist individuals and organizations ensure that employees who are cancer survivors maintain high levels of work engagement. Therefore, it is important for HR practitioners to cultivate employee resilience, workplace social support, and workplace spirituality.

From the cancer survivors' perspective, this study shows that despite their medical conditions, cancer survivors can be as well engaged at the workplace as their healthier co-workers. They generally have similar passions, pride, and joy, and can be as captivated in their work as other healthy employees. Indeed, work empowers cancer survivors to regain normalcy and control over their lives. This also supports the affirmation that many cancer survivors hope to resume work even after being diagnosed with cancer.

## 7. Limitations and future studies

The present study has several limitations. The generalization of the findings across various settings must be considered given the limitation of the sample to a purposively derived group of young adult cancer survivors from one hospital (HKL) and this research is not representative to the cancer survivors in Malaysia, however, the study findings should be further examined and replicated using longitudinal designs that can yield more comprehensive data. Next, this study employed the quantitative research methodology to determine the direct and indirect relationships of employee resilience, workplace social support and quality of working life with work engagement among cancer survivors. The cancer survivors might answer the questions in a manner that was thought would be viewed favorably by others; hence, they might not always be completely truthful. Therefore, to have a better understanding of work engagement from the perspective of cancer survivors, a qualitative study could be conducted in subsequent research. It would be interesting as this kind of research might reveal details based on the individual's experiences relating to work engagement. In addition, through a qualitative approach, a deeper understanding of the phenomenon could be obtained, such as in relation to workplace social support, the present study focusses on collegial and supervisory support, the role of social support in one's private life, such as from a spouse, family and friends should be considered in workplaces by providing opportunities for maintaining a better balance between work and one's private life. Next, the current study uses data from cancer survivors only. Future research could investigate the employer's perspective on cancer- related work outcomes and implementation of interventions based on the employer's experience in managing employees with cancer.

## 8. Conclusion

The findings of this study demonstrate that work-related and work engagement concerns of young adult cancer survivors are currently unexplored. It is evident that work engagement among young adult cancer survivors is complex specifically regarding employee resilience, workplace social support, and workplace spirituality. Hence, one must consider the impact of treatments that young adults with cancer shall encounter when they want to return to work. This study also confirmed that workplace spirituality mediated the influence of employee resilience and workplace social support toward work engagement. This finding is in line with the generally reported significant relationship between employee resilience and workplace social toward workplace spirituality. The findings also showed that workplace social support is a strong predictor of work engagement. It can be surmised that going to work with a positive frame of mind and emotions in the form of spirituality strongly influence cancer survivors to be very much engaged at the workplace. Moreover, the level of employees' work engagement would increase when they find that their jobs bring meaning and purpose to their lives. Moreover, when cancer survivors enjoy workplace social support, there would be a corresponding increase in their level of work engagement.

## Data availability statement

The datasets presented in this article are not readily available because, due to the nature of this research, participants of this study did not agree for their data to be shared publicly, so supporting data is not available. Requests to access the datasets should be directed at: SM, syuhada.musa@upm.edu.my.

## Ethics statement

The studies involving human participants were reviewed and approved by Medical Ethics Committee and National Medical Research Registry (NMRR18-85-40225-IIR) Ministry of Health Malaysia. The patients/participants provided their written informed consent to participate in this study.

## Author contributions

Conceptualization: SM and SH. Methodology, software, formal analysis, data curation, writing—original draft preparation, and visualization: SM. Validation: SM, SH, and ZM. Investigation: SM and ZM. Resources: ZM. Writing—review and editing: SM, SK, and SH. Supervision: SH and SA. Project administration and funding acquisition: SH. All authors have read and agreed to the published version of the manuscript.
